# PEGylated Curcumin Derivative Attenuates Hepatic Steatosis via CREB/PPAR-*γ*/CD36 Pathway

**DOI:** 10.1155/2017/8234507

**Published:** 2017-07-09

**Authors:** Yu Liu, Fei Cheng, Yuxuan Luo, Zhu Zhan, Peng Hu, Hong Ren, Huadong Tang, Mingli Peng

**Affiliations:** ^1^Key Laboratory of Molecular Biology for Infectious Diseases (Ministry of Education), Institute for Viral Hepatitis, Department of Infectious Diseases, The Second Affiliated Hospital, Chongqing Medical University, Chongqing, China; ^2^Zhejiang University of Technology, Hangzhou 310014, China

## Abstract

Curcumin has the potential to cure dyslipidemia and nonalcoholic fatty liver disease (NAFLD). However, its therapeutic effects are curbed by poor bioavailability. Our previous work has shown that modification of curcumin with polyethylene glycol (PEG) improves blood concentration and tissue distribution. This study sought to investigate the role of a novel PEGylated curcumin derivative (Curc-mPEG454) in regulating hepatic lipid metabolism and to elucidate the underlying molecular mechanism in a high-fat-diet- (HFD-) fed C57BL/6J mouse model. Mice were fed either a control chow diet (D12450B), an HFD (D12492) as the NAFLD model, or an HFD with Curc-mPEG454 administered by intraperitoneal injection at 50 mg/kg or 100 mg/kg for 16 weeks. We found that Curc-mPEG454 significantly lowered the body weight and serum triglyceride (TG) levels and reduced liver lipid accumulation in HFD-induced NAFLD mice. It was also shown that Curc-mPEG454 suppressed the HFD-induced upregulated expression of CD36 and hepatic peroxisome proliferator activated receptor-*γ* (PPAR-*γ*), a positive regulator of CD36. Moreover, Curc-mPEG454 dramatically activated cAMP response element-binding (CREB) protein, which negatively controls hepatic PPAR-*γ* expression. These findings suggest that Curc-mPEG454 reverses HFD-induced hepatic steatosis via the activation of CREB inhibition of the hepatic PPAR-*γ*/CD36 pathway, which may be an effective therapeutic for high-fat-diet-induced NAFLD.

## 1. Introduction

Nonalcoholic fatty liver disease (NAFLD) has been recognized as the most common chronic hepatic disease and is characterized by increased triglyceride accumulation as lipid droplets in hepatocytes. Obesity, dyslipidemia, hyperuricemia, and insulin resistance enhance the risk of NAFLD, ranging from simple hepatic steatosis to nonalcoholic steatohepatitis (NASH), advanced fibrosis, and cirrhosis, with prevalence varying from 25% to 40% across different countries [1–3]. At present, there are no approved drugs for the treatment of hepatic lipid metabolism disorder or steatohepatitis by the USA and China Food and Drug Administration [4]. Only OCA (obeticholic acid) has shown promise in improving liver histology, highlighting the urgent need to develop effective therapeutic strategies for NAFLD [5, 6]. Recent advances have focused on several natural polyphenols, such as curcumin, anthocyanins, and resveratrol, which are proposed as promising drug candidates for treatment of NAFLD and possibly future clinical applications [7–9].

Curcumin, a natural polyphenol from turmeric, has been shown to be effective in attenuating liver histopathological changes, reducing cholesterol (TC), triglycerides (TG), and free fatty acid (FFA) levels in the serum and liver, and improving insulin resistance in rodent animals [10–12]. Recently, two randomized double-blind clinical trials, in which NAFLD treatment was performed for 8 weeks, have shown that curcumin significantly lowered the liver fat content, body mass index, and serum levels of TC, TG, low-density lipoprotein cholesterol (LDL-C), glucose, and uric acid compared with the placebo group [13, 14]. These findings suggest that curcumin has the potential to cure dyslipidemia and NAFLD. However, its therapeutic effects are curbed due to poor bioavailability and very fast systemic elimination [15, 16]. Hence, researchers have made great efforts to improve the bioavailability of curcumin. Modification of a drug with polyethylene glycol (PEG) is a well-known technology for improving the physicochemical properties and biological response of a drug [17]. Moreover, PEG modification has been used to overcome the low aqueous solubility and increase the stability of curcumin [18].

**Figure 1:**
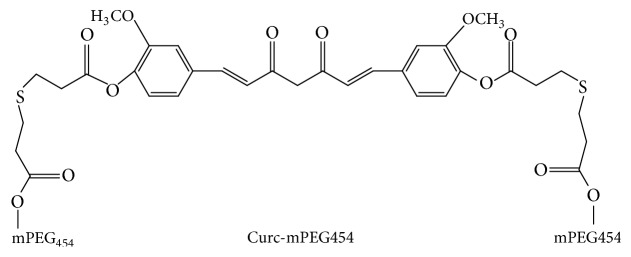
The chemical structure of Curc-mPEG454.

Here, we have produced water-soluble curcumin by conjugating two low-molecular-weight PEGs (mPEG454) via *β*-thioester bonds (Curc-mPEG454, Figure 1), which had a fixed composition with a curcumin-loading content of 25.3% and released curcumin in the liver. The conjugate retained the biological activities of the native curcumin towards cancer [19]. Therefore, it is reasonable to hypothesize that the curcumin component of Curc-mPEG454 could attenuate lipid metabolism and hepatic steatosis. To investigate the lipid-lowering effects of the PEGylated curcumin derivatives and explore the underlying mechanism in treatment of NAFLD, a high-fat-diet (HFD) mouse model was used in this study. Interestingly, we found that Curc-mPEG454 could effectively decrease serum TG levels and attenuate hepatic steatosis via activation of cAMP response element-binding (CREB) protein and subsequent negative inhibition of hepatic-specific peroxisome proliferator activated receptor-*γ* (PPAR-*γ*) and fatty acid transporter (CD36) expression. These findings suggest that Curc-mPEG454 has therapeutic potential in NAFLD by regulating lipid metabolism via the CREB/PPAR-*γ*/CD36 pathway. Our results may direct the use of curcumin for the clinical treatment of NAFLD.

## 2. Materials and Methods

### 2.1. Chemical

Amphiphilic curcumin (Curc-mPEG454) was synthesized by Dr. Huadong Tang. Its characterizations are available online at https://www.futuremedicine.com/toc/nnm/5/6. Chloroform, methanol, formalin, and so forth were obtained from the Center of Equipment and Reagent (Chongqing University of Medical Sciences). All the reagents were of analytical quality.

### 2.2. Animals and Diets

Six-week-old male C57BL/6J mice (Animal Center of Chongqing Medical University, China) were housed in a room at 22°C with a 12-hour light/dark cycle and free access to food and water. After one week of adaption, the mice were randomly divided into four groups of 10 mice each. Control group mice were fed a chow diet (Research Diets, D12450B, 10% cal% fat) and received an intraperitoneal injection of normal saline (NS). The other three groups were fed a high-fat diet (HFD, Research Diets, D12492, 60% cal% fat) supplemented with NS or Curc-mPEG454 (50 or 100 mg/kg) by intraperitoneal injection every other day for 16 weeks. The body weight of each mouse was measured weekly. At the end of the study, the mice were sacrificed after being withdrawn from food for 12 hours. Blood samples were taken by heart puncture to determine the serum biomarkers. The liver was collected and weighed. A portion of liver tissue was either fixed in 4% paraformaldehyde or placed in optimal cutting temperature (OCT) compound (Sakura Finetek, Tokyo, Japan) for histopathological analysis. In addition, the remaining liver tissues were rapidly frozen in liquid nitrogen for gene and protein expression analyses. All animals received humane care that was approved by the Institutional Animal Care and Ethic Committee of Chongqing Medical University.

### 2.3. Biochemical Parameters

Serum was collected after centrifugation at 1000 rpm for 15 minutes. Serum ALT (CH0101201), AST (CH0101202), TC (CH0101003), TG (CH0101151), HDL-C (CH0101161), LDL-C (CH0101162), and FFA (CH0101157) were measured using routine enzymatic assays using commercial kits (Maccura, Chengdu, China) from HITACHI Clinical Analyzer 7600 (Hitachi, Tokyo, Japan) in the Clinical Laboratory, the Second Affiliated Hospital, Chongqing Medical University.

### 2.4. Histopathological Analysis

**Figure 2:**
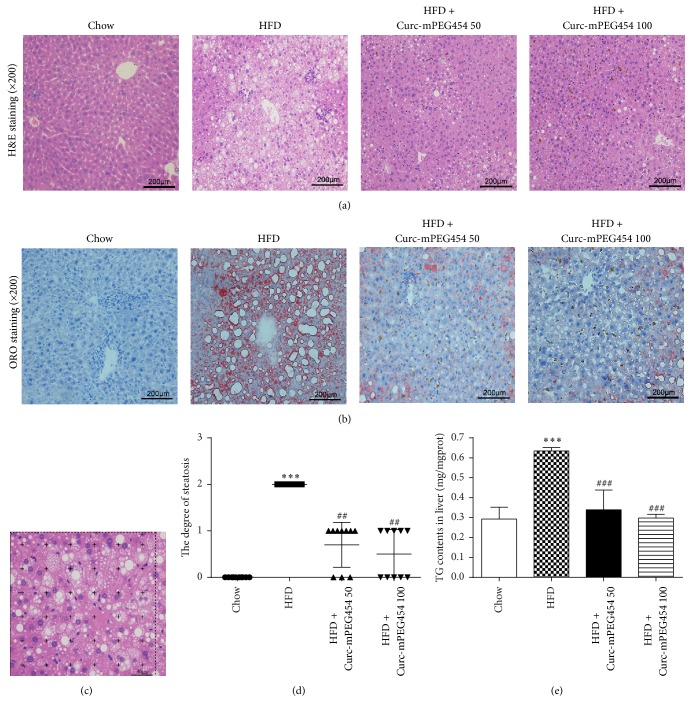
Curc-mPEG454 reduced hepatic steatosis and hepatic lipid levels after 16-week treatment. Compared with the livers in the chow group, HFD-induced typical steatosis was evidenced by H&E staining (a) and oil red O staining (b) of lipids in representative liver section; a test system containing 36 test points was superimposed on HE-stained images, and the degree of steatosis was assessed by point counting (c), the degree of steatosis of each group (d), and the content of liver TG (e). Curc-mPEG454 treatment significantly reversed the changes. Quantified date are mean ± SD. ^*∗∗∗*^*P* < 0.001 versus chow group; ^##^*P* < 0.01 and ^###^*P* < 0.001 versus HFD group.

Liver tissues from the same part of each mouse were fixed in 4% paraformaldehyde, embedded in paraffin, sliced, and stained with hematoxylin and eosin (H&E). Fresh livers were fixed in 4% paraformaldehyde, embedded in OCT, and cut into 10 *μ*m thick sections in a cryostat. Frozen sections were stained with 0.2% oil red O. The image analysis software STEPanizer (University of Bern, Switzerland) was used to analyze HE-stained liver tissue digital images. 36 test points (PT) assembled on the image form the point counting (P-C) method (Figure 2(c)). The percentage of area occupied by liver steatosis (Vv) in the image was measured using P-C method. Vv = click on the proportion of fat vesicles (Pp)/the number of 36 test points (PT) [20]. The degree of steatosis was graded as 0–3 based on the percentage of fat-accumulated hepatocytes per field at 400x magnification under H&E staining (0: none; 1: <33%; 2: 33–66%; and 3: >66%) according to Brunt et al. [21].

### 2.5. Hepatic Lipid Measurement

Liver lipids were extracted using the Folch method [22]. Hepatic triglyceride and cholesterol contents were measured using commercial kits (Jiancheng, Nanjing, China). The concentrations of hepatic TG and TC were analyzed and normalized by protein concentration.

### 2.6. Real-Time PCR and Western Blotting Analysis

Total RNA was extracted from the liver using TRIzol (Invitrogen, USA) according to the manufacturer's instructions. cDNA was synthesized with a commercial kit (Takara, Japan). Gene expression was measured by real-time PCR with CFX Connect™ Real-Time PCR System (Bio-Rad, USA). GAPDH was used as an internal control, and the relative expression levels of mRNA were calculated using the 2^−ΔΔCt^ method. The primer pairs used for real-time PCR are listed in Table 1.

For Western blotting, total protein from liver homogenates was lysed using RIPA containing protease inhibitor cocktail and the phosphatase inhibitor PhosSTOP (Roche, USA). Protein concentration was determined using a BCA Protein Assay Kit (Pierce Chemical, USA). Quantified proteins were separated on SDS-PAGE gels and transferred onto PVDF membranes (Millipore Corporation, USA). After blocking, the membranes were incubated with primary anti-CD36 (1 : 1000, ab133625, Abcam, USA), anti-PPAR-*γ* (1 : 1000, ab41928, Abcam), anti-phospho-CREB (ser133) (1 : 1000, 9198, CST, USA), and anti-CREB (1 : 1000, 9197S, CST) at 4°C overnight. Next, the membrane was washed and incubated with secondary antibodies conjugated to HRP for 2 h at room temperature. *β*-Actin was used as an internal control. Protein bands were visualized using ECL (Pierce Chemical, USA). Levels of target protein band densities were analyzed with the ChemiDoc™ MP Imaging System (Bio-Rad, USA).

### 2.7. Immunohistochemical Analysis of Liver Steatosis

Immunohistochemistry for CD36 and phospho-CREB was performed using the commercial kit (ZSGB-BIO, China) according to the manufacturer's instructions. Photographs were captured by a microscope (Nikon, Japan).

### 2.8. Statistical Analysis

Data were presented as the mean ± standard deviation (SD). Differences between groups were analyzed by one-way ANOVA followed by the Holm-Sidak post hoc test. Statistical significance was considered at *P* values < 0.05.

## 3. Results

### 3.1. Curc-mPEG454 Reduces Body Weight and Plasma TG Level

After 16 weeks of treatment, as shown in Table 2, HFD induced significant elevation of final body weight, body weight gain, and liver weight compared with the chow diet group. Curc-mPEG454 treatment (50 mg/kg and 100 mg/kg) lowered the final body weight and body weight gain compared to the HFD, while there was no dose-dependent difference observed between these two groups. No significant difference in food intake was observed in the four groups, suggesting that the Curc-mPEG454-induced reduction in body weight was not a result of reduced food intake. In addition, we examined the effects of Curc-mPEG454 on serum lipids levels and liver injury. Administration of Curc-mPEG454 significantly lowered plasma TG compared with the HFD but did not affect plasma TC, HLD-C, LDL-C, and FFA levels. Further, no difference in serum ALT and AST among the experimental groups was observed, suggesting that Curc-mPEG454 use was safe and did not induce liver injury in vivo. Taken together, these results illustrate that Curc-mPEG454 effectively lowered plasma TG and corrected for hypertriglyceridemia.

### 3.2. Curc-mPEG454 Attenuates Hepatic Lipid Accumulation

To assess the impact of Curc-mPEG454 on hepatic steatosis induced by HFD, liver sections were stained with H&E and oil red O to measure hepatic lipid accumulation. As shown in Figures 2(a) and 2(b), mice fed the HFD developed severe macrovesicular and microvesicular steatosis without apparent inflammatory cell infiltration and fibrosis, while there appeared to be an attenuated state of steatosis with notable reduction in the number of vacuolar areas in both Curc-mPEG454 treatment groups. We also measured the percentage of steatosis area by analyzing ten H&E sections with STEPanizer in each group and estimated the degree of steatosis according to Burnt et al. (Figure 2(c)). The results show that the degree of steatosis in the HFD group was mainly distributed in grade 2, while it was significantly attenuated to grades 0 and 1 upon Curc-mPEG454 treatment (Figure 2(d)). Consistent with these results, the TG content in the liver was significantly decreased after Curc-mPEG454 supplementation (Figure 2(e)). In contrast, no obvious change in hepatic TC levels was observed in any of the groups (data not shown). These results indicate that Curc-mPEG454 may effectively protect against hepatic steatosis and TG accumulation induced by HFD.

### 3.3. Curc-mPEG454 Inhibits HFD-Induced CD36 Expression

**Figure 3:**
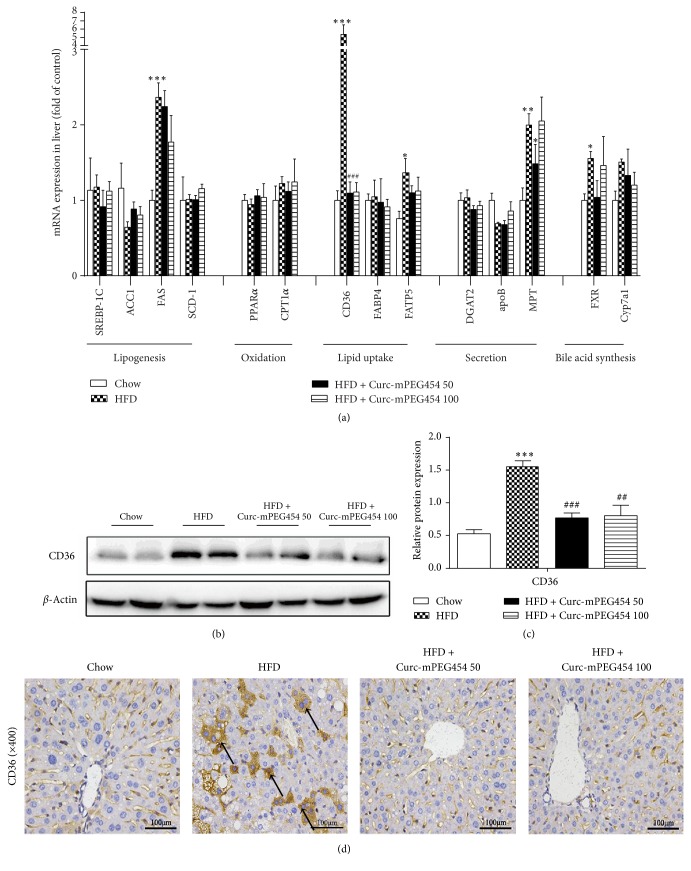
Effects of Curc-mPEG454 on CD36 expression and hepatic lipid metabolic relative genes in mice after 16-week treatment. qPCR analysis of mRNA levels of genes involved in hepatic lipogenesis, FA oxidation, lipid uptake, TG secretion, and bile acid synthesis (a). Western blot analysis and quantification of CD36 protein; *β*-actin served as an internal control ((b) and (c)). The immunohistochemical staining of CD36 in liver tissue; the black arrows indicate positive expression of CD36 (d). All figures are representative of at least 3 independent experiments. Quantified date are mean ± SD. ^*∗*^*P* < 0.05, ^*∗∗*^*P* < 0.01, and ^*∗∗∗*^*P* < 0.001 versus chow group; ^##^*P* < 0.01 and ^###^*P* < 0.001 versus HFD group.

To explore the mechanism underlying the protective effect of Curc-mPEG454 on hepatic steatosis, we assessed for changes in key genes related to lipogenesis, fatty acid uptake, fatty acid oxidation, and TG secretion in liver. Analysis of mRNA expression was presented in Figure 3(a), whereby the mRNA expression of CD36, also named fatty acid transporter (FAT) involved in mediating plasma lipid levels and hepatic fatty acid uptake, was significantly elevated in the HFD and almost restored to normal levels in Curc-mPEG454 (50 and 100 mg/kg) intervention groups. Consistent with observed mRNA expression, the protein level of CD36 was markedly increased with HFD and significantly reduced after treatment with Curc-mPEG454 (50 and 100 mg/kg) (Figures 3(b) and 3(c)), with no significant difference observed between low and high doses of Curc-mPEG454. Immunohistochemistry for CD36 in the liver confirmed that HFD induces higher CD36 membrane exposition of hepatocytes compared to that of the Curc-mPEG454 treated group (Figure 3(d)).

In our study, the level of mRNA expression of genes involved in lipid metabolism, such as sterol regulatory element-binding protein 1C (SREBP-1C), acetyl-CoA carboxylase (ACC1), fatty acid synthase (FAS), and fatty acid transport protein (FATP5), was not affected by HFD treated in the presence or absence of Curc-mPEG454 (Figure 3(a)). These results were inconsistent with a previous study [23] and may be attributed to different diet or drug administration. Since excess liver lipids can be utilized for bile acid synthesis, we examined the expression of genes involved in bile acid synthesis and metabolism. We found that mRNA levels of farnesoid X receptor (FXR) and cholesterol 7-alpha-monooxygenase (Cyp7a1) were unchanged after administration of Curc-mPEG454 (Figure 3(a)). Thus, these results demonstrate that Curc-mPEG454 contributes to improved hepatic steatosis by inhibiting CD36 expression and subsequently reducing FFA uptake and TG synthesis.

### 3.4. Curc-mPEG454 Specifically Suppresses Hepatic PPAR-*γ* Expression

**Figure 4:**
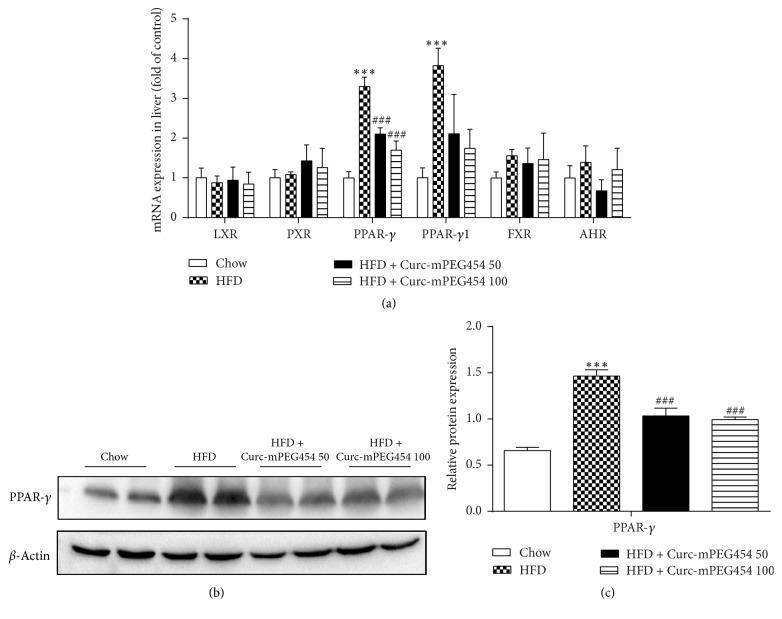
Effects of Curc-mPEG454 on lipid accumulation and PPAR-*γ* expression in liver. qPCR analysis of mRNA levels of genes involved in regulation of CD36 (a). Western blot analysis and quantification of PPAR-*γ* protein; *β*-actin served as an internal control ((b) and (c)). All figures are representative of at least 3 independent experiments. Quantified date are mean ± SD. ^*∗∗∗*^*P* < 0.001 versus chow group; ^###^*P* < 0.001 versus HFD group.

CD36 expression is transcriptionally regulated by several nuclear receptors and transcription factors, such as liver X receptor (LXR), pregnane X receptor (PXR), PPAR-*γ* [24], FXR [25], and aryl hydrocarbon receptor (AHR) [26]. Moreover, we investigated which of these factors may be involved in Curc-mPEG454 treatment. As shown in Figure 4(a), both hepatic PPAR-*γ* and PPAR-*γ*1 were significantly upregulated in the HFD group compared with those in the chow diet group. In addition, the downregulation of PPAR-*γ* and PPAR-*γ*1 occurred after Curc-mPEG454 treatment and is consistent with the change in CD36 liver expression. Moreover, there was no significant dose-dependent difference between low-dose and high-dose Curc-mPEG454-treated groups. Interestingly, no evident changes in other regulators, including LXR, PXR, FXR, and AHR, were observed. PPAR-*γ* plays an important role in lipid storage processes, and its role in the activation of lipogenic genes may contribute to the development of hepatic steatosis. Consistently, Western blot analysis showed that Curc-mPEG454 significantly suppressed PPAR-*γ* protein expression induced by HFD feeding (Figures 4(b) and 4(c)). Consequently, these results suggest that Curc-mPEG454 preferentially and prominently downregulated the elevated hepatic PPAR-*γ* expression levels observed in HFD-fed mice, which then resulted in suppressed CD36 expression.

### 3.5. Curc-mPEG454 Activates CREB Phosphorylation

**Figure 5:**
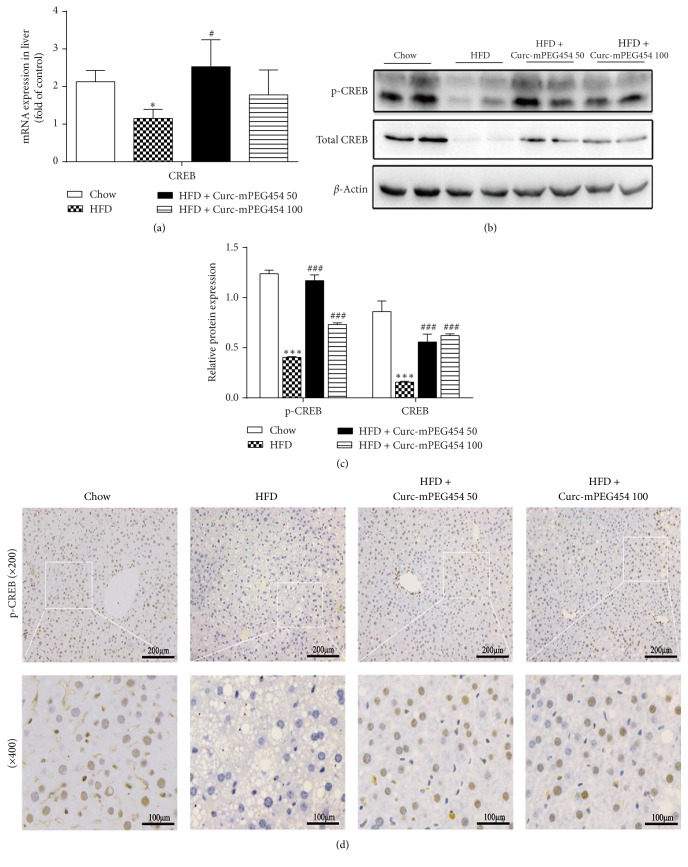
Curc-mPEG454 activated CREB phosphorylation in liver. qPCR analysis of CREB expression (a). Western blot analysis and quantification of phospho-CREB and CREB protein; *β*-actin served as an internal control ((b) and (c)). The immunohistochemical staining of phospho-CREB in liver tissue (d). All figures are representative of at least 3 independent experiments. Quantified data are the mean ± SD. ^*∗*^*P* < 0.05 and ^*∗∗∗*^*P* < 0.001 versus chow group; ^#^*P* < 0.05 and ^###^*P* < 0.001 versus HFD group.

CREB is an upstream molecule that regulates PPAR-*γ* expression [27] and the cAMP/protein kinase A (PKA)/CREB signaling pathway plays an important role in hepatic lipid metabolism [28]. Therefore, we wondered whether the regulatory effects of Curc-mPEG454 on hepatic steatosis may be via activation of the CREB/PPAR-*γ* pathway. We found that mRNA and protein expression of CREB in the liver was dramatically downregulated by high-fat diet. Interestingly, Curc-mPEG454 treatment increased both total and phosphorylated CREB levels regardless of the dose used (Figures 5(a), 5(b), and 5(c)). Immunohistochemical studies demonstrated that Curc-mPEG454 treatment activates the immunoreactivity of p-CREB (Figure 5(d)). Hence, our data suggest that Curc-mPEG454 may coordinate hepatic lipids by activating CREB phosphorylation, resulting in the inhibition of PPAR-*γ*.

## 4. Discussion

Curcumin improves hepatic lipid metabolism and plasma lipid homeostasis, which consequently lowers the risk of hepatic inflammation and steatosis, diabetes, insulin resistance, and atherosclerosis. However, the poor bioavailability of curcumin compromises its clinical applications. Therefore, improvement of curcumin bioavailability is crucial to its use in clinical applications. Pandey et al. demonstrated that PEG modification can improve the aqueous solubility and bioavailability of curcumin [29]. In this study, we adopted Curc-mPEG454 modified with low-molecular-weight PEG. Previous studies have demonstrated that Curc-mPEG454 produced approximately 50–500 times higher serum curcumin levels than orally, intravenously, or intraperitoneally administered curcumin [19]. In the current study, we found that the mice treatment with Curc-mPEG454 at dose of 50 mg/kg could achieve similar lipid-lowering effects as 1000 mg/kg curcumin treatment in Zingg's research [28]. Therefore, we speculate that PEGylation improves the pharmacokinetics and pharmacodynamics of curcumin. Moreover, Curc-mPEG454 exhibits better bioactivity than parent curcumin. We also found that there was no difference between the two doses of Curc-mPEG454 used in our study. Therefore, we speculate that Curc-mPEG454 at dose of 50 mg/kg has reached an effective serum concentration and that increasing the drug dose will not enhance its curative effects.

The imbalance between FFA input and output in the liver leads to TG deposition and hepatic steatosis. The major sources of FFA in the liver were diet supplementation, de novo synthesis in the liver, and adipose tissue decomposition. The massive FFA in liver can be metabolized via *β*-oxidation in mitochondria, esterified to formation of TG and storage in lipid droplets, or combined with apolipoproteins and secreted into blood through VLDL [30]. In this study, we found that Curc-mPEG454 treatment significantly downregulated HFD-induced hepatic CD36 expression and then reduced the hepatic FFA uptake from the peripheral circulation FFA. Moreover, we observed no impact on genes involved in lipogenesis, beta-oxidation, TG secretion, and bile acid metabolism. CD36 functions as a transporter mediating liver long-chain FFA uptake and esterification, which regulates the development of hepatic steatosis [31]. Clinical studies have shown that dysregulated expression of hepatic CD36 was significantly associated with insulin resistance, hyperinsulinemia, and increased steatosis in patients with NAFLD and chronic hepatitis C [32]. Elevated expression of CD36 was also observed in mouse models with genetic obesity and high-fat-feeding-induced fatty livers [33]. Therefore, therapeutic strategies aimed at reversing this process by restoring normal CD36 levels may provide a new approach to therapy for NAFLD.

CD36 is a shared target of LXR, PXR, PPAR-*γ* [24], FXR [25], and AHR [26] when mediating lipid homeostasis. Nuclear receptors and transcription factors establish a cross-talk network regulating the homeostasis of bile acids, lipid, glucose, inflammation, vitamins, hormones, and others [34]. In the present study, we prove that Curc-mPEG454 specifically reduces the expression of hepatic PPAR-*γ*, which is originally described as a transcriptional factor crucial for controlling lipid homeostasis [35, 36]. Furthermore, hepatocyte-specific PPAR-*γ* expression is positively associated with fatty liver in mouse models. For instance, overexpression of PPAR-*γ* leads to hepatic steatosis and hepatocyte-specific knockout of PPAR-*γ* reduces hepatic fat content in HFD-fed mice [37, 38]. Taken together, the protective effects of Curc-mPEG454 against hepatic steatosis are possibly via specific inhibition of PPAR-*γ*/CD36 pathway activation and subsequent reduction of FFA uptake and TG synthesis in the liver.

**Figure 6:**
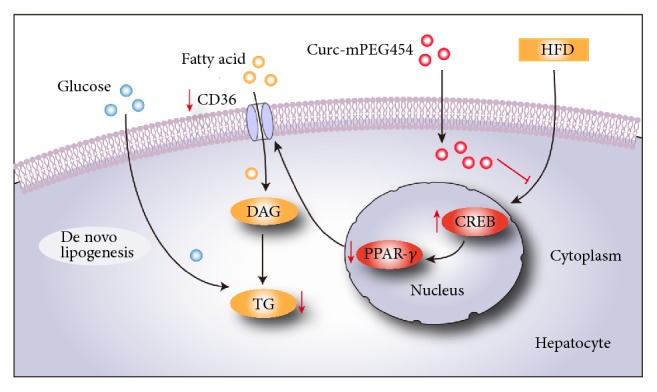
Schematic illustration of molecular mechanism involved in antisteatosis of Curc-mPEG454. The red arrows indicate that Curc-mPEG454 may coordinate hepatic lipid through activation of CREB phosphorylation and then inhibition of PPAR-*γ* and CD36 expression. The pathway of CREB/PPAR-*γ*/CD36 plays a crucial role in lipid metabolism in HFD-induced NAFLD.

CREB, an upstream molecular target of PPAR-*γ*, is a transcription factor that plays an important role in gluconeogenesis and fatty acid oxidation [39–41]. Herzig's research found that CREB inhibits hepatic TG synthesis and storage during fasting via PPAR-*γ* repression and demonstrated that mice infected with a dominant-negative CREB-expressing adenovirus progressed into fatty liver with increased hepatic TG content consistent with elevated hepatic PPAR-*γ* and CD36 expression [27]. Inoue et al. reported that CREB expression in liver was significantly suppressed by high-fat diet and CREB/PPAR-*γ* signaling pathways may be involved in HFD-induced hepatic steatosis [35]. A recent study revealed that curcumin may contribute to its hypolipidemic effect by increasing cAMP levels and the phosphorylation of CREB in LDL^−/−^ mice fed an HFD [28]. Based on these findings, we raise a hypothesis that Curc-mPEG454 controls hepatic metabolism through CREB negative regulation of PPAR-*γ*. Our results showed that Curc-mPEG454 dramatically activated CREB phosphorylation consistent with decreased PPAR-*γ*. Hence, we speculate that activation of the hepatic CREB/PPAR-*γ*/CD36 pathway by Curc-mPEG454 is crucial for reducing lipid accumulation in liver (Figure 6).

In summary, our study demonstrates that Curc-mPEG454 possesses protective effects against dyslipidemia and fatty liver, similar to curcumin. In addition, our findings provide new insight into the mechanism underlying curcumin-mediated fatty liver. Hence, PEGylated curcumin has better bioavailability and can be used as a promising candidate for NAFLD therapy.

## Figures and Tables

**Table 1:** Primers to analyze murine genes by real-time PCR.

Genes	Accession number	Forward primer	Reverse primer
GAPDH	GU214026	5′-TGACGTGCCGCCTGGAGAAA-3	5′-AGTGTAGCCCAAGATGCCCTTCAG-3′
SREBP-1C	NM_001313979.1	5′-GCACACACCCAGAACTTGC-3′	5′-GCACCACCAACTGCCACTAT-3′
ACC1	XM_006531957.3	5′-TGCCAATCTCATTTCCTCCT-3′	5′-TTTCTTCCTTCGCCTCCTTT-3′
FAS	NM_007988.3	5′-TGGGTTCTAGCCAGCAGAGT-3′	5′-ACCACCAGAGACCGTTATGC-3′
SCD-1	NM_009127.4	5′-TTCTTGCGATACACTCTGGTGC-3′	5′-CGGGATTGAATGTTCTTGTCGT-3′
PPAR*α*	NM_001113418.1	5′-GTGGGTGGTTGAATCGTGAG-3′	5′-GCAGTGGAGTTTGGGTTGG-3′
CPT1*α*	NM_013495.2	5′-ACGTTGGACGAATCGGAACA-3′	5′-GGTGGCCATGACATACTCCC-3′
CD36	NM_031561.2	5′-TGGTCAAGCCAGCTAGAAA-3′	5′-CCCAGTCTCATTTAGCCAC-3′
FABP4	NM_024406.2	5′-AAGGTGAAGAGCATCATAACCC-3′	5′-TCACGCCTTTCATAACACATTCC-3′
FATP5	NM_009512.2	5′-TCGGATCTGGGAATTCTACG-3′	5′-TTGGTTCTTTCGAACCTTGG-3′
DGAT2	NM_026384.3	5′-GTGGTCAGCAGGTTGTGTGT-3′	5′-CAAGAAAGGTGGCAGGAGAT-3′
apoB	NM_009693.2	5′-TTCCCGTGTTCCAATCAAAT-3′	5′-CCTCCACCAAACTGCTCTTC-3′
MPT	NM_008642.2	5′-ATCATCATTGGAGCCCTGGT-3′	5′-CATTCTTCAGGGCCAGCA-3′
Cyp7a1	NM_007824.2	5′-GGCATTTGGACACAGAAGCATAGA-3′	5′-ACTCGGTAGCAGAAGGCATACAT-3′
LXR	EU869275	5′-TGAAGGCGTCCACCATTGAGAT-3′	5′-GGCGATAAGCAAGGCATACTCTG-3′
PXR	AF031814	5′-CGGAGAAGACGGCAGCATCT-3′	5′-CAGGTGTGGCAGAAGAGGGAT-3′
PPAR-*γ*	NM_001127330	5′-GCTCCACACTATGAAGACATTCCA-3′	5′-CCACAGACTCGGCACTCAATG-3′
PPAR-*γ*1	AB644275.1	5′-GGAAGACCACTCGCATTCCTT-3′	5′-GTAATCAGCAACCATTGGGTCA-3′
FXR	*NM_009108.2*	5′-GGCCTCTGGGTACCACTACA-3′	5′-ACATCCCCATCTCTTTGCAC-3′
AHR	NM_001314027.1	5′-ACCAGAACTGTGAGGGTTGG-3′	5′-TCTGAGGTGCCTGAACTCCT-3′
CREB	NM_001037726.1	5′-TGCCACCTTGCCTGAGACTG-3′	5′-ATGAGCCTGCCTTCCACTTGAT-3′

The primers were designed with Primer 6 and verified by Oligo 7.

**Table 2:** Effect of Curc-mPEG454 on body, liver weight, serum lipids, ALT, and AST in high-fat-diet-fed mice.

Group	Chow	HFD	HFD + Curc-mPEG454 50	HFD + Curc-mPEG454 100
Original body weight (g)	21.30 ± 1.16	21.13 ± 0.99	21.38 ± 1.19	20.55 ± 1.01
Final body weight (g)	30.81 ± 2.06	44.08 ± 5.44^*∗∗*^	37.64 ± 3.13^#^	37.67 ± 4.36^#^
Body weight gain (g)	9.51 ± 2.45	22.95 ± 5.14^*∗∗*^	16.26 ± 3.42^#^	17.12 ± 4.56^#^
Liver weight (g)	1.06 ± 0.09	1.43 ± 0.28^*∗*^	1.35 ± 0.21	1.27 ± 0.19
Serum TG (mmol/L)	0.67 ± 0.05	0.84 ± 0.06^*∗*^	0.63 ± 0.12^##^	0.61 ± 0.06^##^
Serum TC (mmol/L)	2.82 ± 0.06	4.17 ± 0.52^*∗*^	3.72 ± 0.85	3.44 ± 0.87
Serum HDL-C (mmol/L)	1.22 ± 0.07	1.77 ± 0.19^*∗*^	1.68 ± 0.24^*∗*^	1.66 ± 0.36^#^
Serum LDL-C (mmol/L)	0.39 ± 0.06	0.41 ± 0.06	0.32 ± 0.08	0.28 ± 0.09^#^
Serum FFA level (mmol/L)	1.05 ± 0.15	1.07 ± 0.13	1.03 ± 0.14	1.19 ± 0.16
Serum ALT level (mmol/L)	53.75 ± 8.57	55.25 ± 10.9	40.25 ± 12.66	43.75 ± 9.43
Serum AST level (mmol/L)	128.25 ± 17.80	128.75 ± 19.98	121.00 ± 18.65	112.00 ± 13.14

Body weight gain was calculated by the difference between final body weight at the end of the experiments and original body weight. Data are expressed as mean ± SD. ^*∗*^*P* < 0.05 versus chow and ^*∗∗*^*P* < 0.01 versus chow; ^#^*P* < 0.05 versus HFD and ^##^*P* < 0.01 versus HFD.

## Abbreviations

ACC1: Acetyl-CoA carboxylase
AHR: Aryl hydrocarbon receptor
ALT: Alanine aminotransferase
AST: Aspartate aminotransferase
CREB: cAMP response element-binding
FATP5: Fatty acid transport protein
FAS: Fatty acid synthase
FFA: Free fatty acid
FXR: Farnesoid X receptor
H&E: Hematoxylin and eosin
HDL-C: High-density lipoprotein cholesterol
HFD: High-fat diet
LDL-C: Low-density lipoprotein cholesterol
LXR: Liver X receptor
NAFLD: Nonalcoholic fatty liver disease
NASH: Nonalcoholic steatohepatitis
PEG: Polyethylene glycol
PKA: cAMP/protein kinase A
PPAR-*γ*: Peroxisome proliferator activated receptor-*γ*
PXR: Pregnane X receptor
SREBP-1C: Sterol regulatory element-binding protein 1C
TC: Cholesterol
TG: Triglycerides.
